# Comparative Analysis of Volatile Composition in Chinese Truffles via GC × GC/HR-TOF/MS and Electronic Nose

**DOI:** 10.3390/ijms17040412

**Published:** 2016-04-05

**Authors:** Ning Zhang, Haitao Chen, Baoguo Sun, Xueying Mao, Yuyu Zhang, Ying Zhou

**Affiliations:** 1 College of Food Science and Nutritional Engineering, China Agricultural University, Beijing 100083, China; zh_ningts@163.com (N.Z.); wumaoxy@163.com (X.M.); 2 Beijing Laboratory for Food Quality and Safety, Beijing Technology and Business University (BTBU), Beijing 100048, China; 3 Beijing Advanced Innovation Center for Food Nutrition and Human Health (BTBU), Beijing 100048, China; 4 Beijing Key Laboratory of Flavor Chemistry, Beijing Technology and Business University (BTBU), Beijing 100048, China; zhangyy2@163.com; 5 Yunnan ZhuoYi Food Company LTD, Jiangchuan 650032, China; Zyzhouying.666@163.com

**Keywords:** GC × GC/HR-TOF/MS, electronic nose, DSE-SAFE, Chinese truffles

## Abstract

To compare the volatile compounds of Chinese black truffle and white truffle from Yunnan province, this study presents the application of a direct solvent extraction/solvent-assisted flavor evaporation (DSE-SAFE) coupled with a comprehensive two-dimensional gas chromatography (GC × GC) high resolution time-of-flight mass spectrometry (HR-TOF/MS) and an electronic nose. Both of the analytical methods could distinguish the aroma profile of the two samples. In terms of the overall profile of truffle samples in this research, more kinds of acids were detected via the method of DSE-SAFE. Besides, compounds identified in black truffle (BT), but not in white truffle (WT), or *vice versa*, and those detected in both samples at different levels were considered to play an important role in differentiating the two samples. According to the analysis of electronic nose, the two samples could be separated, as well.

## 1. Introduction

The species of truffle (*Tuber* F. H. Wigg.) occur mostly in the Northern Hemisphere, and they are distributed across Asia, Europe, North Africa and North America [1]. They are fungi belonging to the truffle genus (Ascomycota), which form below-ground ascocarps or fruiting bodies (hypogeous fungi) [2]. Among edible fungi, truffle may represent the best known and most expensive ones in commercial value due to their organoleptic qualities. It is well known that *T. melanosporum* Vittad., the Périgord black truffle from France, and *T. magnatum* Pico., the Piedmont white truffle from Italy, are considered as the most valuable species.

Truffles are abundant in some regions of China, especially in the southwest. However, it was not until the 1980s that research on truffles in China started [3]. During the past three decades, more truffle species were discovered in China [4], and this indicated that edible truffle diversity is much richer in natural resources than expected. In China, truffles can adapt to a wide range of soil conditions, and they are mainly associated with forest woods, like *Pinus. yunnanensis* and *P. armandii*, *etc.* [5]. In these woods, most truffle species are harvested from November to the following March.

About 200 volatiles in total have been reported in the literature for the entire truffle species investigated from different areas of the world. However, there was a clear distinction between Chinese and European truffle [6,7]. The common methods for truffle volatile extraction included headspace analysis, dynamic headspace, purge-and-trap and stir bar sorptive extraction (SBSE) [6,8,9] and headspace solid-phase microextraction (HS-SPME) [10,11], coupled with gas chromatography/mass spectrometry (GC/MS), comprehensive two-dimensional gas chromatography/flame ionization detector/mass spectrometry (GC × GC-(FID/MS)) [12] and an electronic nose [13] as a method of discrimination, as well.

In this research, a comparison of volatile compounds between the two kinds of Chinese truffle, black and white ones, had been established in terms of qualitative and semi-quantitative differences on volatile compounds. The method of direct solvent extraction/solvent-assisted flavor evaporation (DSE-SAFE) coupled with comprehensive two-dimensional gas chromatography/high resolution time-of-flight mass spectrometry (GC × GC/HR-TOF/MS) was applied to identify aroma compounds. Meanwhile, the study also characterized the overall aroma profiles of samples by an electronic nose.

## 2. Results

### 2.1. Comprehensive Two-Dimensional gas Chromatography (GC × GC)/High Resolution Time-of-Flight Mass Spectrometry (HR-TOF/MS) Analysis

**Figure 1:**
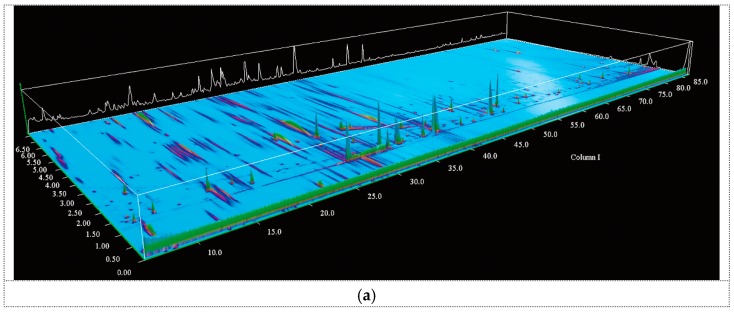

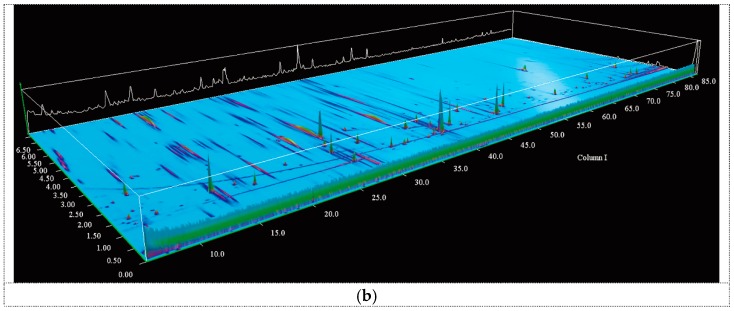
The 3D chromatogram image of volatiles detected by comprehensive two-dimensional gas chromatography (GC × GC) high resolution time-of-flight mass spectrometry (HR-TOF/MS): (**a**) black truffle (BT); (**b**) white truffle (WT).

**Figure 2:**
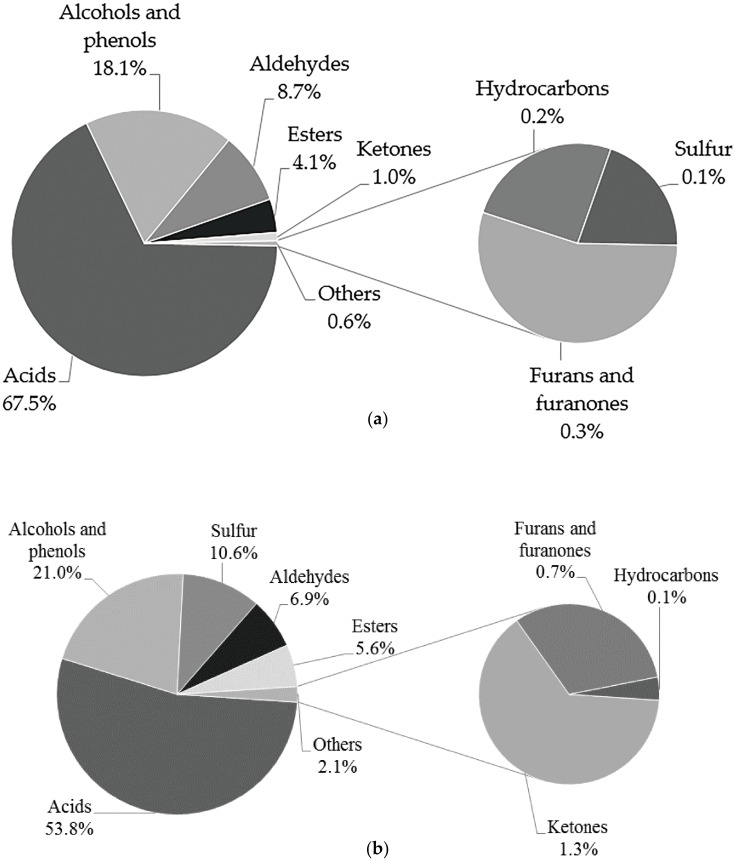
Variability of the compositions in fruiting bodies of BT (**a**) and WT (**b**) based on concentrations.

As shown in Figure 1, the volatile compounds of the black truffle (BT) and white truffle (WT) samples were separated and identified using a DB-Wax (polyethylene glycol) column on the first dimension and a DB-5 (5% phenyl/methylpolysiloxane) column on the second dimension via GC × GC/HR-TOF/MS analysis. The volatile (71 in total) compounds found in the samples are shown in Table 1, and they were classified into eight groups. From Table 1, it could be seen that differences existed between the two samples on the basis of the identified compositions. A total of 58 volatile components, including 14 alcohols and phenols, 13 aldehydes, 2 hydrocarbons, 6 ketones, 10 acids, 6 esters, 5 furans and furanones and 2 sulfur-containing compounds, were identified in black truffles. In contrast, a total of 47 volatile components, comprising 9 alcohols and phenols, 12 aldehydes, 1 hydrocarbon, 3 ketones, 10 acids, 3 esters, 4 furans and furanones and 5 sulfur-containing compounds, were found in the white samples. For the sake of differentiating the overall profiles more clearly, each group of volatiles was expressed as the percentage of the total compositions, and the results of both samples are displayed in Figure 2. The profile of both samples was dominated by acids (67.5% in BT *vs.* 53.9% in WT), alcohols and phenols (18.1% in BT *vs.* 20.9% in WT), aldehydes (8.7% in BT *vs.* 6.9% in WT) and esters (4.1% in BT *vs.* 5.7% in WT). Besides, the proportion of sulfur-containing volatiles presented a notable variation with 0.1% in BT and 10.7% in WT.

Notwithstanding the differences of the aromatic profile presented above, the two samples also shared some common features. According to Table 1, some volatile compositions (34 in total) were found in both of the truffle samples, which accounted for 47.9% of the total aroma compounds. The identical compounds included 7 alcohols and phenols, 10 aldehydes, 1 hydrocarbon, 2 ketones, 8 acids, 2 esters, 3 furans and furanones and 1 sulfur-containing compound. In terms of the contents performed, it was notable that 3-methyl-butanoic acid (No. 44), hexanoic acid (No. 47), phenylethyl alcohol (No. 10) and 2-methyl-1-butanol (No. 3) were abundant in both BT and WT, whereas 2-methylpropanoic acid (No. 42) was only abundant in BT and benzyl benzoate (No. 58) in WT. Moreover, hexanal (No. 18), benzeneacetaldehyde (No. 28), benzaldehyde (No. 27), 2,4-di-tert-butylphenol (No. 15), 1-octen-3-ol (No. 8) and acetoin (No. 38) were also present at relatively higher levels in both samples, while 5-ethylcyclopent-1-enecarboxaldehyde (No. 25), (*E*)-2-octenal (No. 26) and 1-pentanol (No. 4) were more abundant in BT.

**Figure 3:**
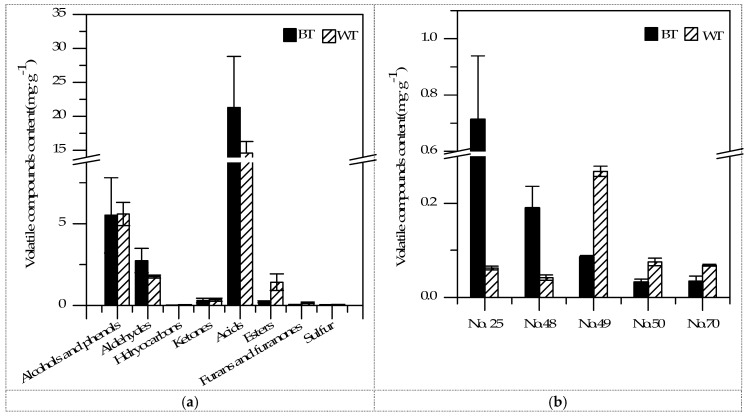
Contents of volatile compounds detected in BT and WT: (**a**) volatiles classified by chemical families; (**b**) volatiles with a significance value lower than 0.05 (No. 25: 5-ethylcyclopent-1-enecarboxaldehyde; No. 48: heptanoic acid; No. 49: octanoic acid; No. 50: nonanoic acid; No. 70: 3-(methylthio)propanoic acid).

**Figure 4:**
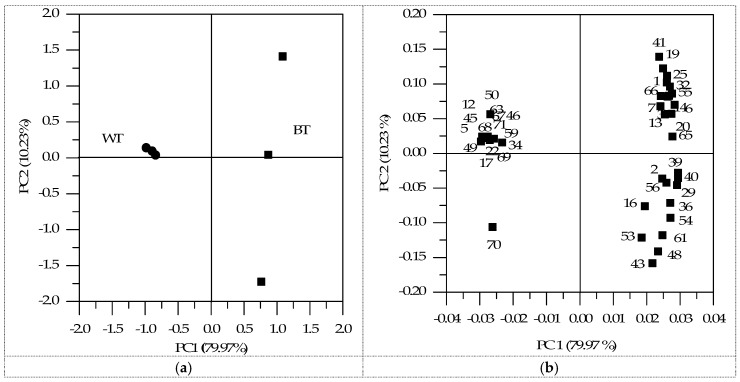
Principle component analysis (PCA) score plot: (**a**) BT and WT samples; (**b**) the compounds detected in both samples with a significant difference and those detected in BT, but not in WT, or *vice versa* (numbers correspond to Table 1).

As shown in Figure 3, although the same components were identified, they were distinguished in terms of contents. Besides, according to the outcomes using *t*-tests, five kinds of compounds showed significant differences between BT and WT in terms of the concentration, and the results are shown in Figure 3. The volatiles included 1 aldehyde, 3 acids and 1 sulfur-containing compound. In addition, it should be noted that 24 kinds of compounds were identified only in BT, while 13 compounds only in WT. In general, the compounds detected in both samples at different levels together with those detected in BT, but not in WT, or *vice versa*, must play an important role in distinguishing the overall volatile profiles of the two samples. The corresponding aroma description and threshold values of these compounds (42 in total) are shown in Table 2. Besides, the PCA of these compounds was performed based on their concentrations, and the results are shown in Figure 4. The two principle components (PC1 and PC2) accounted for 90.20% of the whole variance (79.97% and 10.23%, respectively). The PCA result of the two samples showed that BT and WT could be clearly separated on the PCA plot. It could be seen that BT was located on the positive side of the PC1 dimension and kept separate from the sample cluster of WT. Additionally, the major compounds contributing to the positive dimension of the PC1 component included dodecanal (No. 29), acetophenone (No. 39), benzophenone (No. 40), 1-hexanol (No. 6), (*E*)-9-octadecenoic acid ethyl ester (No. 55) and dibenzofuran (No. 65). In contrast, major compounds contributing to the negative dimension of the PC1 component included octanoic acid (No. 49), 3-methyl-1-pentanol (No. 5), *β*-ethylphenethyl alcohol (No. 12), 3-methyl-2-butenal (No. 22), 3-methyl-2-butenoic acid (No. 45), 4-methylpentanoic acid (No. 46), furyl hydroxymethyl ketone (No. 63), methional (No. 67), (methylthio)-cyclohexane (No. 68), (*Z*)-2-butenal (No. 17) and 3-methylthio-1-propanol (No. 69). Moreover, the important compounds on the positive side of PC2 included propanoic acid (No. 41), 2-methyl-2-butenal (No. 19), 5-ethylcyclopent-1-enecarboxaldehyde (No. 25) and 2-butanol (No. 1). For the negative side of PC2, butanoic acid (No. 43), heptanoic acid (No. 48), isobornyl acetate (No. 53), dihydro-5-methyl-2(3*H*)furanone (No. 61) and 3-(methylthio)propanoic acid (No. 70) were the main contributors.

It can be seen that the compounds identified in BT, but not in WT, showed higher values on the positive side of PC1, while the same tendency appeared in WT on the negative side. Therefore, these compositions must play an active role in distinguishing the two samples, especially for those discussed above that contribute to the PC1 dimension greatly. Furthermore, for the compounds detected in both samples at different levels, it was obvious that 5-ethylcyclopent-1-enecarboxaldehyde (No. 25) and heptanoic acid (No. 48) were more sensitive to BT on the positive side of PC1, while octanoic acid (No. 49), nonanoic acid (No. 50) and 3-(methylthio)propanoic acid (No. 70) were more sensitive to WT on the negative side of PC1. Moreover, heptanoic acid (No. 48) and 3-(methylthio)propanoic acid (No. 70) were distinguished from the other three compounds on the PC2 dimension. In general, differences in the composition of volatile components from BT and WT were observed, and these data could be used for the discrimination of each sample.

### 2.2. Electronic Nose Response

**Figure 5:**
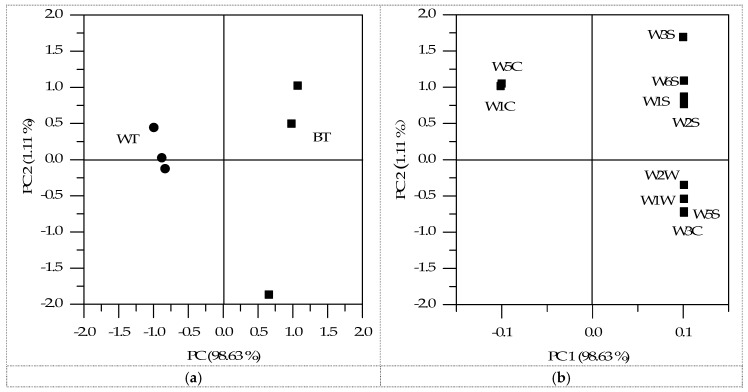
Principle component analysis (PCA) results of electronic nose analysis: (**a**) BT and WT samples; (**b**) ten sensors (W1C, W5S, W3C, W6S, W5C, W1S, W1W, W2S, W2W and W3S) of electronic nose.

The response signals of ten sensors (ratio of conductance, *G/G_0_*) were close to 1.0 in the initial period and then changed continuously until they stabilized after approximately 50 s. The response signals of each sensor at 56 to 58 s were used in the subsequent analyses. Principle component analysis (PCA) was performed, and the cross-validation was used to investigate the difference of the two samples. The PCA results are shown in Figure 5. According to electronic nose analysis, PCA allowed the samples to be easily separated in score plots by combining PC1 (98.63%) with PC2 (1.11%). It was obvious that BT was located on the positive of the PC1 dimension and kept apart from the cluster of WT on the negative side. Considering the sensors used in the electronic nose, the eight sensors, W5S, W3C, W6S, W1S, W1W, W2S, W2W and W3S, were more sensitive to BT on the positive side of the PC1 dimension. In contrast, the sensors of W1C and W5C were more sensitive towards WT on the negative side of PC1. It could be seen that different sensors in the array were sensitive to the diverse volatile compounds released from BT and WT. In general, BT and WT could be distinguished according to the PCA analysis of the electronic nose.

## 3. Discussion

### 3.1. GC × GC/HR-TOF/MS Analysis

The alcohols, aldehydes and ketones, produced by lipid oxidation and Strecker degradation of amino acids [9], remarkably contributed to the truffle flavor. For instance, 1-octen-3-ol (along with other C8 volatiles) was a potential signal molecule produced by both truffle mycelium and fruiting bodies [20,21], which had been found in most truffles. 1-Pentanol was once detected in both *T. borchii* and *T. melanosporum* [6]. 2-Methyl-1-butanol has previously been reported as a volatile compound in *T. magnatum*. Phenylethyl alcohol has also been identified in some kinds of truffle species, such as *T. aestivum* [22] and *T. mesentericum* [23]. A series of short chain aldehydes, such as hexanal, heptanal, (*Z*)-2-heptenal, nonanal, 5-ethylcyclopent-1-enecarboxaldehyde, *E*-2-octenal, benzaldehyde, benzeneacetaldehyde, 2,4-decadienal and α-ethylidene-benzeneacetaldehyde, were identified in both samples, and all of them were found in various species of truffle [6,10]. Acetoin, previously discovered in *T. melanosporum* (Soria) [10] and *T. indicum* [6], has a bland, woody, yogurt odor and a strong buttery and creamy aroma when diluted to 1.0%.

According to the published literature, acids detected in truffle volatile components seemed fewer. Though identified, there were only a small number of acids, such as acetic acid, 2-propenoic acid and 2-methylhexanoic acid [10,23]. A total of twelve acid compounds were identified in this research, which dominated the aroma profile of both samples with the maximum proportion (67.5% in BT, 53.9% in WT) based on concentrations. Meanwhile, eight acids were detected in both of the samples: 2-methylpropanoic acid, 3-methyl-butanoic acid, hexanoic acid, heptanoic acid, octanoic acid, nonanoic acid, (*E*)-2-octenoic acid and benzeneacetic acid. All of the “extraction systems” have different performances linked to their different chemical and physical characteristics. In terms of the different reported methods applied to extract truffle volatiles, the widely-used methods, SPME and SBSE, are very efficient via divinylbenzene/carboxen/polydimethylsiloxane fiber (DVB/CAR/PDMS; thickness: 50/30 µm). Besides, SPME is a powerful technique for the analysis of volatile organic sulfur compounds in truffle aromas [24]. DSE can extract most of the components in the samples, including volatile and nonvolatile compounds based on the theory of “like dissolves like”. When combined with SAFE, this method allows the fast and careful isolation of volatiles from solvent extracts of food [25] and is able to remove any nonvolatile materials. The method DSE-SAFE has been used in many aspects of volatile compounds in food, such as fruit [26], milk [27], sauces [28], meat [29], wines [30], and so on. However, the method acts as a new technique in the detection of volatiles in truffles.

Considering furans and furanones detected in the samples, 2-pentylfuran has been detected in *T. excavatum*, *T. aestivum* [23] and *T. aestivum*, *T. melanosporum* [10] via SPME. It has an odor of fruity, green and earthy beany with a vegetable-like nuance. Both dihydro-5-pentyl-2(3*H*) furanone (coconut, creamy, waxy, sweet, buttery and oily) and 2(5*H*) furanone (buttery) had been found in *T. melanosporum* [6] by SBSE.

Both aroma profiles and the amount of volatile compounds from truffles varied in species [31]. According to Table 1, in addition to the identical composition with various concentrations, the difference between the two samples was also reflected in the types of compounds identified, while 24 of them were only detected in BT samples and 13 of them only in WT samples.

Among the 30–60 volatiles produced by a single truffle, only a small amount of them contributes to the aroma profile [32]. Besides, sulfur-containing compounds were essential compositions, for they acted as truffle aromas that humans perceived. In this study, more sulfur-containing compounds were detected in white truffles in terms of both amounts and contents. In accordance with the results, sulfur-containing volatiles in black truffle (0.040 µg·g^−1^ in total) included dimethyl disulfide and 3-(methylthio) propanoic acid, while compounds in white truffle (2.894 µg·g^−1^ in total) included methional, (methylthio)-cyclohexane, 3-methylthio-1-propanol, 3-(methylthio) propanoic acid and benzothiazole. The diversity of sulfur-containing volatiles will surely play an important role in the formation of aromatic profiles due to their low olfactory threshold, as well as to distinguish the samples [24]. Dimethyl disulfide has been identified in many truffles to date [7,33]. Methional and benzothiazole were found in some kinds of *T.* *magnatum* species, but varied according to geographical areas [34]. (Methylthio)-cyclohexane has been reported in anchovy sauce [35]. 3-Methylthio-1-propanol was detected in *T. borchii* and *T. melanosporum*, as reported via SBSE [6], which has a powerful, sweet, soup or meat-like odor and flavor in high dilution. 3-(Methylthio)propanoic acid was once identified in fresh pulp of pineapple [36], but there was no relevant literature about it in truffle by far. Therefore, further determination still needs to be made to confirm whether it actually came from the fresh truffle or was generated during transportation or storage. In general, the various concentration of volatile compounds led to the formation of different aroma profiles of the two samples.

### 3.2. Electronic Nose Response

The PCA results reduced the dimensionality of the original data and compared all of the variables with the same normalized standard distribution [37] by explaining the correlation among those underlying factors without losing much information. Factor score plots were used to indicate similar, dissimilar and typical data. The PCA analysis was carried out based on the sensor signals of the volatile gases of BT and WT. According to the PCA results, all of the sensors were sensitive to the volatile gases of the samples on PC1 component.

## 4. Materials and Methods

### 4.1. Chemicals and Samples

Two truffle samples, both black and white ones, were obtained from Kunming, Yunnan province, China. In this research, BT and WT were used to represent the black truffle and white truffle samples, respectively. Truffle samples was harvested with the best quality according to the experience of farmers and free from any quality deterioration or decay. Immediately after harvest, the ascocarps were wrapped with aluminum foil paper, vacuumized and sealed in vacuum packing bags. Afterwards, the samples were transported to our laboratory by air (ice bags were used during transportation) and stored at −20 °C for further preprocessing within a month. For each kind of truffle, all of the ascocarps were cut up, being fully mixed and thawed at room temperature (20 ± 2 °C) for about 1 h right before the extracting procedure, respectively.

The solvent, diethyl ether (AR), was purchased from Sinopharm Chemical Reagent Co., Ltd. (Shanghai, China) and was redistilled before used. The solvent, methanol, and the internal standard, 1,2-dichlorobenzene, were of HPLC grade. The methanol was from Thermo Fisher Scientific Inc. (Shanghai, China) and the internal standard was from Aladdin Industrial Corporation (Shanghai, China). For the measurement of retention indices (RI), a mix of n-alkanes ranging from hexane to triacontane was used (Sigma-Aldrich Co., St. Louis, MO, USA).

### 4.2. Extraction Methods

#### 4.2.1. Direct Solvent Extraction

Twenty grams of freshly-cut dices of truffle sample were weighed and put into an erlenmeyer flask. Distilled water (100 mL) and 50 µL of an internal standard stock solution (1.3 μg·μL^−1^ of 1,2-dichlorobenzene) were added to the flask, saturated with analytical-grade sodium chloride. The mixture was homogenized at 1500 rpm for 5 min using an SCILOGEX *BlueSpin* LED digital hotplate magnetic stirrer (MS-H280-Pro, Berlin, CT, USA). The samples were treated through an extraction procedure with 100 mL diethyl ether in a flask. During extraction, the mixtures were agitated for 30 min at 1500 rpm at room temperature (20 ± 2 °C). Then, the solvent phase (upper layer) was separated by centrifugation at 3000 rpm for 10 min at 4 °C. The procedure was repeated another two times, and then, the solvent extract (300 mL in total) was obtained.

#### 4.2.2. Solvent-Assisted Flavor Evaporation

Extracts prepared by DSE were subjected to SAFE to remove any nonvolatile materials via SAFE [24,38]. The technique is operated by connecting a compact to a distillation vessel for a rapid and high yield isolation of volatiles from solvent extracts. A high vacuum is applied in the apparatus to isolate volatiles from the organic phase. After removal of non-volatile compounds, the SAFE distillate was dried over anhydrous Na_2_SO_4_ and then was slowly concentrated to 10 mL using a rotatory evaporator. Final volumes of extracts were concentrated using a gentle stream of nitrogen gas to 500 µL. Samples were prepared in triplicate and stored in 2-mL glass vials at −85 °C for GC × GC/HR-TOF/MS analysis.

### 4.3. GC × GC/HR-TOF/MS Analysis

The Fas TOF GC × GC/HR-TOF/MS system consisted of an Agilent 7890 (Agilent Technologies, Palo Alto, CA, USA) gas chromatograph, a cold-jet modulator and a high resolution time-of-flight mass spectrometer (Zoex Corp., Lincoln, NE, USA), which had a high scan rate up to 500 Hz, about 3500 resolution and high sensitivity. The data processing software was GC-image HRMS 2.3 ((Zoex Corp.). The first column was DB-Wax (15 m × 0.25 mm i.d. (inner diameter) × 0.25 µm film thickness), and the second column was DB-5 (2.8 m × 0.1 mm i.d. × 0.1 µm film thickness); both were purchased from Agilent. A volume of 1 µL of the sample was injected into the GC injector with a split ratio of 20:1 at 250 °C. The modulation period was 7 s, and the hot jet widths were 300 ms. Separation was performed by using the following temperature program: initial temperature 40 °C, ramped at 2 °C/min to 145 °C and held for 5 min, then ramped at 3 °C/min to 230 °C and held for 2 min. The transfer line into the TOF-MS source was heated at 280 °C, and the electron impact ionization source operated at 230 °C with a collision energy of 70 eV. The data acquisition rate was 100 Hz over a mass range of 30 to 500 amu.

### 4.4. Compounds Identification

The identification of volatiles of the truffle samples was based on an NIST11 library search. The mass spectral match factor (similarity > 700) was used to judge whether a peak was correctly identified or not. For the determination of the retention index (RI), calculated on the first dimension (DB-Wax column), a series of n-alkanes (C_6_ to C_30_) were used under the same experimental conditions. Semi-quantitative data of the aroma compounds were calculated by relating the peak areas of volatiles to the peak area of the internal standard (1,2-dichlorobenzene) [39].

The computational formula of RI is as follows:

(1) $$\text{RI}=100\times \left\{n+\frac{\mathrm{lg}t\prime_{\left(i\right)}-\mathrm{lg}t\prime_{\left(n\right)}}{\mathrm{lg}t\prime_{\left(n+1\right)}-\mathrm{lg}t\prime_{\left(n\right)}}\right\}$$

where *n* and (*n +* 1) are respectively the number of carbon atoms in alkanes eluting before and after the compound, *t′(n)* and *t′(n* + 1*)* are the corresponding retention time and *t′(i)* is the retention time of the compound to be identified (*t′(n) < t′(i)* *< t′(n* + 1*)*).

### 4.5. Electronic Nose Analysis

An electronic nose device PEN 3 E-Nose was used, which was manufactured by Winmuster Airsense Analytic Inc., Schwerin, Germany. The sensor array system consisted 10 metal oxide semiconductors (MOS) of different chemical compositions and thicknesses to provide selectivity towards volatile compound classes, including W1C (aromatic compounds), W5S (broad-range compounds), W3C (ammonia, aromatic compounds), W6S (hydrogen), W5C (aromatic-aliphatic), W1S (methane, broad-range compounds), W1W (sulfur compounds), W2S (broad-alcohol compounds), W2W (sulfur-chlorine), and W3S (methane-aliphatic). The software of the electronic nose, Winmuster, was used for data storage and multivariate statistical processing [40].

Two grams of BT and WT samples were put into 15-mL airtight vials (concentration chamber), respectively. The samples were prepared in a mild water bath (50 ± 2 °C) for 50 min, and then, one Luer-lock needle (20 g) connected to a Teflon-tubing (3 mm) was used to perforate the seal (plastic) of the vial and to absorb the volatile gases inside it. Clean air to replace the sampled air was furnished through a second needle connected to a charcoal filter. During the measurement time (60 s), the sampling unit inhaled the volatile gases present in the headspace at a constant rate causing changes in the sensor’s conductance, which was long enough for the sensor signals to reach a steady value. When a measurement was completed, a standby of 500 s was initiated with the circuit, and the chamber was flushed by clean air until the sensor signals returned to baseline. The E-Nose analysis was performed at least three times for each truffle sample.

### 4.6. Statistical Analysis

Results were expressed as the mean ± standard deviation (SD) of at least three independent pretreatment experiments (extracting procedure) for each sample. The experimental results of the categories for the volatile compounds were performed using *t*-tests. Statistical significance was determined at *p* < 0.05. The principal component analysis (PCA) was also performed against the differences of volatile compounds between BT and WT samples. All statistical analyses were performed using the SPSS-software package.

## 5. Conclusions

In general, this research demonstrated the method DSE-SAFE coupled with GC × GC/HR-TOF/MS and an electronic nose applied to conduct comparative analysis of volatile compositions in Chinese black truffle and white truffle from Yunnan province. The differences were shown in both of the analytical results. In terms of the overall profile of truffle samples in this research, more kinds of acids were detected via the method of DSE-SAFE. According to the PCA analysis results of GC × GC/HR-TOF/MS, compounds identified in BT, but not in WT, or *vice versa*, especially those contributing much to the PC1 component, and the compounds detected in both samples at different levels were considered to play an effective role in distinguishing the two samples. Moreover, BT and WT could also be distinguished according to electronic nose analysis. In the future, further studies will be executed to investigate the aroma compounds of Chinese truffles, as well as the application of its unique flavor in the modern process of the food industry in China.

## Figures and Tables

**Table 1:** Volatile compounds identified in black truffle (BT) and white truffle (WT) via direct solvent extraction/solvent-assisted flavor evaporation (DSE-SAFE) combined with comprehensive two-dimensional gas chromatography (GC × GC) high resolution time-of-flight mass spectrometry (HR-TOF/MS).

^a^ No.	^b^ RI Exp	^b^ RI Lit	Compound Name	^c^ CAS No.	Library Match Factor	Black Truffle (BT)		White Truffle (WT)		BT	WT
						^d^ RT I (min)	^d^ RT II (s)	RT I (min)	RT II (s)	^e^ Mean ± SD (µg·g^−1^)	Mean ± SD (µg·g^−1^)
**Alcohols and Phenols**											
1	1010	1016	2-Butanol	78-92-2	925	7.09	1.39	ND	ND	0.032 ± 0.011	ND
2	1070	1078	2-Methyl-1-propanol	78-83-1	883	9.26	1.43	ND	ND	0.266 ± 0.090	ND
3	1188	1206	2-Methyl-1-butanol	137-32-6	879	14.56	1.64	14.63	1.63	1.695 ± 0.852	2.541 ± 0.235
4	1231	1241	1-Pentanol	71-41-0	893	16.97	1.66	16.92	1.66	0.144 ± 0.028	0.069 ± 0.001
5	1333	1323	3-Methyl-1-pentanol	589-35-5	872	ND	ND	22.81	1.88	ND	0.092 ± 0.010
6	1334	1345	1-Hexanol	111-27-3	871	22.88	1.87	ND	ND	0.032 ± 0.007	ND
7	1410	1430	1-Methoxy-3-methyl benzene	100-84-5	907	27.39	2.77	ND	ND	0.137 ± 0.058	ND
8	1431	1442	1-Octen-3-ol	3391-86-4	893	28.59	2.22	28.49	2.24	0.218 ± 0.048	0.339 ± 0.035
9	1470	1481	2-Ethyl-1-hexanol	104-76-7	881	30.92	2.40	30.91	2.40	0.082 ± 0.024	0.051 ± 0.004
10	1875	1875	Phenylethyl Alcohol	60-12-8	934	53.19	1.65	53.16	1.64	3.100 ± 1.264	1.956 ± 0.215
11	1880	1902	Butylated Hydroxytoluene	128-37-0	887	53.43	5.21	53.36	5.21	0.017 ± 0.001	0.099 ± 0.041
12	1950	1950	*β*-Ethylphenethyl alcohol	2035-94-1	811	ND	ND	57.51	2.15	ND	0.024 ± 0.003
13	2057	2068	3-Methylphenol	108-39-4	803	63.32	1.30	ND	ND	0.033 ± 0.012	ND
14	2106	2107	2-Phenoxyethanol	122-99-6	777	65.61	1.49	ND	ND	0.017 ± 0.005	ND
15	2286	2277	2,4-Di-tert-butylphenol	96-76-4	914	72.49	1.85	72.39	1.86	0.255 ± 0.082	0.543 ± 0.175
16	2556	1779	α-Methylbenzeneethanol	698-87-3	780	80.61	1.34	ND	ND	0.121 ± 0.061	ND
**Aldehydes**											
17	1017	1037	(*Z*)-2-Butenal	15798-64-8	883	ND	ND	7.33	1.54	ND	0.097 ± 0.021
18	1054	1051	Hexanal	66-25-1	906	8.77	2.54	8.70	2.53	0.716 ± 0.198	0.523 ± 0.005
19	1067	1088	2-Methyl-2-butenal	1115-11-3	893	9.14	1.95	ND	ND	0.010 ± 0.004	ND
20	1105	1128	(*E*)-2-Pentenal	1576-87-0	861	10.58	1.93	ND	ND	0.004 ± 0.001	ND
21	1160	1184	Heptanal	111-71-7	865	13.24	3.30	13.09	3.33	0.033 ± 0.014	0.019 ± 0.004
22	1170	1200	3-Methyl-2-butenal	107-86-8	791	ND	ND	13.74	1.92	ND	0.008 ± 0.001
23	1294	1319	(*Z*)-2-Heptenal	57266-86-1	912	20.59	2.90	20.51	2.91	0.099 ± 0.021	0.032 ± 0.003
24	1368	1387	Nonanal	124-19-6	837	24.81	4.71	24.70	4.73	0.047 ± 0.010	0.031 ± 0.008
25	1384	1404	5-Ethylcyclopent-1-enecarboxaldehyde	36431-60-4	882	25.78	3.19	25.71	3.19	0.714 ± 0.225 ^a^	0.062 ± 0.004 ^b^
26	1400	1416	(*E*)-2-Octenal	2548-87-0	876	26.74	3.34	26.68	3.35	0.244 ± 0.074	0.061 ± 0.002
27	1486	1478	Benzaldehyde	100-52-7	896	31.81	1.93	31.76	1.94	0.223 ± 0.047	0.320 ± 0.014
28	1598	1622	Benzeneacetaldehyde	122-78-1	927	38.60	1.96	38.53	1.97	0.568 ± 0.119	0.551 ± 0.031
29	1682	1700	Dodecanal	112-54-9	861	42.90	5.97	ND	ND	0.200 ± 0.010	ND
30	1775	1767	2,4-Decadienal	2363-88-4	837	47.89	3.10	47.84	3.10	0.024 ± 0.005	0.014 ± 0.001
31	1887	1907	α-Ethylidene-benzeneacetaldehyde	4411-89-6	852	53.80	2.35	53.76	2.35	0.076 ± 0.022	0.151 ± 0.016
**Hydrocarbons**											
32	1003	1008	α-Pinene	80-56-8	913	6.84	6.44	ND	ND	0.040 ± 0.012	ND
33	2276	2322	Fluorene	86-73-7	824	72.05	2.35	72.02	2.35	0.011 ± 0.001	0.023 ± 0.017
**Ketones**											
34	—	1012.6	2-Methyl-3-pentanone	565-69-5	776	ND	ND	6.24	2.26	ND	0.005 ± 0.002
35	1000	1016	3-Methyl-2-pentanone	565-61-7	852	6.72	2.24	6.73	2.24	0.014 ± 0.003	0.019 ± 0.007
36	1103	1108	(*E*)-3-Penten-2-one	3102-33-8	849	10.46	1.83	ND	ND	0.002 ± 0.000	ND
37	1158	1175	2-Heptanone	110-43-0	867	13.12	3.14	ND	ND	0.007 ± 0.002	ND
38	1255	1271	Acetoin	513-86-0	836	18.30	1.42	18.37	1.42	0.292 ± 0.128	0.337 ± 0.078
39	1605	1607	Acetophenone	98-86-2	768	38.96	2.07	ND	ND	0.015 ± 0.001	ND
40	2431	2443	Benzophenone	119-61-9	772	76.99	1.99	ND	ND	0.014 ± 0.000	ND
**Acids**											
41	1513	1508	Propanoic acid	79-09-4	935	33.46	1.17	ND	ND	0.654 ± 0.304	ND
42	1540	1544	2-Methylpropanoic acid	79-31-2	939	35.10	1.33	35.22	1.20	3.808 ± 1.026	0.155 ± 0.021
43	1598	1613	Butanoic acid	107-92-6	874	38.60	1.22	ND	ND	1.005 ± 0.381	ND
44	1643	1647	3-Methyl-Butanoic acid	503-74-2	835	40.89	1.37	40.83	1.32	11.014 ± 4.253	10.281 ± 1.507
45	1771	1776	3-Methyl-2-butenoic acid	541-47-9	861	ND	ND	47.60	1.22	ND	0.008 ± 0.001
46	1780	1803	4-Methylpentanoic acid	646-07-1	819	ND	ND	48.08	1.29	ND	0.013 ± 0.002
47	1821	1816	Hexanoic acid	142-62-1	909	50.30	1.37	50.26	1.33	5.247 ± 1.878	3.109 ± 0.143
48	1929	1934	Heptanoic acid	111-14-8	879	56.25	1.46	56.18	1.45	0.190 ± 0.046 ^a^	0.042 ± 0.006 ^b^
49	2041	2038	Octanoic acid	124-07-2	877	62.52	1.44	62.35	1.45	0.086 ± 0.003 ^a^	0.268 ± 0.011 ^b^
50	2150	2144	Nonanoic acid	112-05-0	847	67.30	1.45	67.19	1.46	0.032 ± 0.007 ^a^	0.075 ± 0.008 ^b^
51	2166	2182	(*E*)-2-Octenoic acid	1871-67-6	868	68.03	1.34	67.91	1.34	0.037 ± 0.011	0.049 ± 0.002
52	2544	2543	Benzeneacetic acid	103-82-2	895	80.25	1.21	80.25	1.20	0.891 ± 0.258	0.628 ± 0.036
**Esters**											
53	1550	1550	Isobornyl acetate	125-12-2	798	35.67	5.76	ND	ND	0.019 ± 0.011	ND
54	2235	2241	Hexadecanoic acid ethyl ester	628-97-7	842	70.60	7.13	ND	ND	0.031 ± 0.005	ND
55	2453	2476	(*E*)-9-Octadecenoic acid ethyl ester	6114-18-7	897	77.67	6.09	ND	ND	0.400 ± 0.106	ND
56	2502	2515	9,12-Octadecadienoic acid ethyl ester	7619-08-1	904	79.04	5.33	ND	ND	0.710 ± 0.174	ND
57	2502	2510	Linoleic acid ethyl ester	544-35-4	801	79.08	6.53	78.92	5.32	0.037 ± 0.016	0.051 ± 0.029
58	2581	2607	Benzyl benzoate	120-51-4	914	81.33	2.06	81.33	2.07	0.184 ± 0.042	1.373 ± 0.474
59	1966	1978	Dehydromevalonic lactone	2381-87-5	891	ND	ND	58.52	1.70	ND	0.113 ± 0.032
**Furans and Furanones**											
60	1209	1215	2-Pentylfuran	3777-69-3	915	15.65	4.75	15.51	4.80	0.020 ± 0.004	0.013 ± 0.001
61	1566	1589	Dihydro-5-methyl-2(3*H*)furanone	108-29-2	780	36.67	1.63	ND	ND	0.041 ± 0.011	ND
62	1711	1712	2(5*H*)furanone	497-23-4	861	44.39	1.34	44.33	1.34	0.020 ± 0.007	0.124 ± 0.032
63	1960	1984	Furyl hydroxymethyl ketone	17678-19-2	730	ND	ND	58.12	1.49	ND	0.013 ± 0.001
64	1985	2003	Dihydro-5-pentyl-2(3*H*)furanone	104-61-0	846	59.58	2.53	59.57	2.53	0.014 ± 0.004	0.029 ± 0.004
65	2203	2270	Dibenzofuran	132-64-9	801	69.47	2.30	ND	ND	0.015 ± 0.003	ND
**Sulfur**											
66	1047	1039	Dimethyl disulfide	624-92-0	751	8.41	2.05	ND	ND	0.006 ± 0.003	ND
67	1421	1429	Methional	3268-49-3	897	ND	ND	28.01	1.81	ND	0.900 ± 0.076
68	1650	1684	(Methylthio)-cyclohexane	7133-37-1	705	ND	ND	41.07	2.18	ND	0.609 ± 0.069
69	1691	1710	3-Methylthio-1-propanol	505-10-2	906	ND	ND	43.24	1.50	ND	1.310 ± 0.249
70	2281	2298	3-(Methylthio)propanoic acid	646-01-5	817	72.29	1.16	72.14	1.16	0.034 ± 0.011 ^a^	0.068 ± 0.002 ^b^
71	1906	1936	Benzothiazole	95-16-9	701	ND	ND	54.85	2.15	ND	0.007 ± 0.002

^a^ Volatile compounds were listed in order of the chemical group; ^b^ The retention indices of compounds on the DB-Wax column calculated against the GC × GC/HR-TOF/MS retention time of n-alkanes (C_6_ to C_30_). “Exp”: experimentally-measured on the first column (DB-Wax). “Lit”: retention index (van den Dool and Kratz, 1963 [14]) reported in the literature DB-Wax GC column or equivalents from NIST11; ^c^ “CAS”: Chemical Abstracts Service; ^d^ “RT I” means the retention time (min) of compounds on the first dimension. “RT II” means the retention time (s) of compounds on the second dimension; ^e^ The content of compounds was calculated by the internal standard quantitatively identified by DSE-SAFE combined with GC × GC/HR-TOF/MS, and the data were the “mean standard deviation”. Data in the same row with different superscript letters are significantly different (*p* < 0.05) (statistical analysis was performed using *t*-tests). “ND” means not detected.

**Table 2:** Aroma description and threshold values of the major volatile compounds in BT and WT with a significant difference and the compounds that can be detected in BT, but not in WT, or *vice versa*.

No. ^a^	Compound	Aroma Threshold Values	Description
1	2-Butanol	1700 ppb ^b^	—
2	2-Methyl-1-propanol	360 ppb to 3.3 ppm ^c^	A penetrating, wine-like, disagreeable odor ^c^
5	3-Methyl-1-pentanol	830 ppb to 1.2 ppm ^c^	A fruity, green, slightly pungent odor ^c^
6	1-Hexanol	200 ppb to 2.5 ppm ^c^	An herbaceous, woody, fragrant, mild, sweet, green, fruity odor ^c^
7	1-Methoxy-3-methyl benzene	^d^	—
12	β-Ethylphenethyl alcohol	—	—
13	3-Methylphenol	650 ppb ^b^	A dry, tarry, medicinal-leathery odor ^c^
14	2-Phenoxyethanol	—	—
16	α-Methylbenzeneethanol	—	—
17	(*Z*)-2-Butenal	—	—
19	2-Methyl-2-butenal	—	—
20	(*E*)-2-Pentenal	—	fruity, strawberry ^e^
22	3-Methyl-2-butenal	—	an almond odor ^c^
25	5-Ethylcyclopent-1-enecarboxaldehyde	—	—
29	Dodecanal	0.5 to 1.5 ppb ^c^	A characteristic fatty odor reminiscent of violet on dilution ^c^
32	α-Pinene	2.5 to 62 ppb ^c^	A characteristic odor of pine; it is turpentine-like ^c^
34	2-Methyl-3-pentanone	—	—
36	(*E*)-3-Penten-2-one	—	—
37	2-Heptanone	1 ppb to 1.33 ppm ^c^	A fruity, spicy, cinnamon, banana, slightly spicy odor ^c^
39	Acetophenone	170 ppb ^c^	A characteristic sweet, pungent and strong medicinal odor ^c^
40	Benzophenone	—	A delicate, persistent, rose-like odor ^c^
41	Propanoic acid	5 to 10 ppm ^c^	A pungent, rancid odor ^c^
43	Butanoic acid	240 ppb to 4.8 ppm ^c^	A persistent, penetrating, rancid, butter-like odor ^c^
45	3-Methyl-2-butenoic acid	—	A green, phenolic, dairy aroma ^c^
46	4-Methylpentanoic acid	810 ppb ^c^	an unpleasant, sour, penetrating odor ^c^
48	Heptanoic acid	640 ppb to 10.4 ppm ^c^	A disagreeable rancid, sour, sweat-like, fatty odor ^c^
49	Octanoic acid	910 ppb to 19 ppm ^c^	A mildly unpleasant odor ^c^
50	Nonanoic acid	3 to 9 ppm ^c^	A fatty, characteristic odor ^c^
53	Isobornyl acetate	—	A pleasant, camphor-like odor reminiscent of some varieties of pine needles ^c^
54	Hexadecanoic acid ethyl ester	2 ppm ^c^	A mild, waxy sweet odor ^c^
55	(*E*)-9-Octadecenoic acid ethyl ester	—	—
56	9,12-Octadecadienoic acid ethyl ester	—	—
59	Dehydromevalonic lactone	—	—
61	Dihydro-5-methyl-2(3*H*)furanone	—	A sweet, herbaceous odor ^c^
63	Furyl hydroxymethyl ketone	—	—
65	Dibenzofuran	—	Rotten, rubber, fat, moss ^e^
66	Disulfide dimethyl	0.16 to 1.2 ppb ^c^	A diffuse, intense onion odor ^c^
67	Methional	0.02 ppb ^c^	A powerful, onion, meat-like odor
68	(Methylthio)-cyclohexane	—	—
69	3-Methylthio-1-propanol	0.2 ppb ^c^	A powerful, sweet, soup or meat-like odor and flavor in high dilution ^c^
70	3-(Methylthio)propanoic acid	—	—
71	Benzothiazole	80 to 450 ppb ^c^	A delicate, persistent, rose-like odor similar to that of quinoline ^c^

^a^ Numbers correspond to Table 1; ^b^ Shimoda, M. *et al.* 1996 [15]; ^c^ Georgea A. Burdock. 2010 [16]. Ong, P.K.C. *et al.* 1998 [17]; ^d^ Threshold values/description not available; ^e^ Jordán, M. J. *et al.* 2002 [18]; Varlet, V. *et al.* 2006 [19].
